# Trimethylamine-N-oxide (TMAO)-induced atherosclerosis is associated with bile acid metabolism

**DOI:** 10.1186/s12944-018-0939-6

**Published:** 2018-12-19

**Authors:** Lin Ding, Mengru Chang, Ying Guo, Lingyu Zhang, Changhu Xue, Teruyoshi Yanagita, Tiantian Zhang, Yuming Wang

**Affiliations:** 1 College of Food Science and Engineering, Ocean University of China, No.5 Yushan Road, Qingdao, Shandong Province 266003 People’s Republic of China; 2 Qingdao National Laboratory for Marine Science and Technology, Laboratory of Marine Drugs & Biological products, Qingdao, 266237 People’s Republic of China; 3 Department of Applied Biochemistry and Food Science, Laboratory of Nutrition Biochemistry, Saga University, Saga, 840-8502 Japan

**Keywords:** Trimethylamine-N-oxide, Atherosclerosis, Bile acid profiles, Bile acid metabolism

## Abstract

**Background:**

Recently, trimethylamine-N-oxide (TMAO) plasma levels have been proved to be associated with atherosclerosis development. Among the targets aimed to ameliorating atherosclerotic lesions, inducing bile acid synthesis to eliminate excess cholesterol in body is an effective way. Individual bile acid as endogenous ligands for the nuclear receptor has differential effects on regulating bile acid metabolism. It is unclear whether bile acid profiles are mechanistically linked to TMAO-induced development of atherosclerosis.

**Methods:**

Male apoE^−/−^ mice were fed with control diet containing 0.3% TMAO for 8 weeks. Aortic lesion development and serum lipid profiles were determined. Bile acid profiles in bile, liver and serum were measured by liquid chromatographic separation and mass spectrometric detection (LC-MS). Real-time PCRs were performed to analyze mRNA expression of genes related to hepatic bile acid metabolism.

**Results:**

The total plaque areas in the aortas strongly increased 2-fold (*P* < 0.001) in TMAO administration mice. The levels of triglyceride (TG), total cholesterol (TC), low-density lipoprotein cholesterol (LDL-c) in TMAO group were also significantly increased by 25.5% (*P* = 0.044), 31.2% (*P* = 0.006), 28.3% (*P* = 0.032), respectively. TMAO notably changed bile acid profiles, especially in serum, the most prominent inductions were tauromuricholic acid (TMCA), deoxycholic acid (DCA) and cholic acid (CA). Mechanically, TMAO inhibited hepatic bile acid synthesis by specifically repressing the classical bile acid synthesis pathway, which might be mediated by activation of small heterodimer partner (SHP) and farnesoid X receptor (FXR).

**Conclusions:**

These findings suggested that TMAO accelerated aortic lesion formation in apoE^−/−^ mice by altering bile acid profiles, further activating nuclear receptor FXR and SHP to inhibit bile acid synthesis by reducing *Cyp7a1* expression.

## Background

Atherosclerosis is one of the most important causes of death and disability throughout the world. Based on careful clinical researches, seven prominent contributory causes (risk factors) for atherosclerosis have been identified: increased serum cholesterol and blood pressure, diabetes, obesity, a positive family history, smoking, and an atherogenic diet [1]. Recent clinical studies have suggested a correlation between elevated plasma trimethylamine N-oxide (TMAO) levels and atherosclerosis [2–5]. It has been shown that TMAO may exacerbate inflammatory reactions of vascular wall, induce reactive oxygen species production, and impair cholesterol reverse transport, which are involved in development of atherosclerosis [6]. Koeth et al. also showed that TMAO modulated cholesterol and sterol metabolism to promote the progress of atherosclerosis [4].

Bile acids synthesis from cholesterol is the predominant pathway for eliminating excess cholesterol in the body, which contribute to the regression of atherosclerosis [7]. It has been demonstrated that bile acid act as the endogenous ligands for the nuclear receptor farnesoid X receptor (FXR), regulating the activity of genes involved in bile acids synthesis, transport, conjugation and excretion [8]. Individual bile acid has differential effects on bile acid signaling in mice, and the activities of individual bile acids vary markedly under physiological and pathophysiological conditions [9]. Studies have shown that tauromuricholic acid (TMCA) is FXR antagonist [10], and unconjugated bile acids (such as chenodeoxycholic acid (CDCA), lithocholic acid (LCA), deoxycholic acid (DCA) and cholic acid (CA)) act as high­affinity ligand agonists of FXR [9, 11]. The activation of FXR by bile acids downregulates the expression of Cyp7a1 to limit the synthesis of bile acids in the liver through a feedback mechanism [12]. Therefore, the composition of bile acid pool plays a key role in cholesterol homoeostasis. In the present study, the aim was to find the association between TMAO-induced atherosclerosis and bile acid metabolism. The bile acid profiles in bile, liver, and serum were examined in apoE^−/−^ mice fed with TMAO. Real-time PCRs were performed to analyze mRNA expression of genes related to hepatic bile acid metabolism.

## Methods

### Animals and diets

Male apoE^−/−^ mice (C57/BL6 background) aged 9 weeks were purchased from Nanjing qingzilan Co. Ltd. (Nanjing, China). Mice were housed in an air-conditioned room with a 12 h light/dark cycle, a constant temperature of 23 ± 2 °C, and relative humidity of 65 ± 15%. All protocols and procedures were according to the guidelines of ethical committee of experimental animal care at College of Food Science and Engineering, Ocean University of China. Mice were randomly divided into two groups, control group and TMAO group (*n* = 8) fed with control diet containing 0.3% TMAO (Sigma, St. Louis, MO, USA), for 8 weeks. The composition of the diets is shown in Table 1. At the end of the experimental period, mice were sacrificed after a 12 h overnight fasting. Serum was collected from blood by centrifugation at 4000 g at 4 °C for 10 min and was then stored at − 80 °C. Fresh tissue samples were fixed for histopathology determinations or were quick-frozen with liquid nitrogen.

**Table 1:** Experimental diet composition (g/kg)

Ingredient	Control	TMAO
Corn starch	650	650
Casein	150	147.04
Corn oil	50	50
Cellulose	50	50
Mineral mix^a^	35	35
Vitamin mix^b^	10	10
DL-Methionine	3	3
Choline bitartrate	2	2
TMAO	0	2.96

^a^AIN-93 M mineral mix

^b^AIN-93 M vitamin mix

### Atherosclerotic lesion quantitation and histologic analysis

After perfusion with cold PBS (pH, 7.4) and 4% paraformaldehyde, the entire aorta was rapidly dissected from the proximal ascending aorta to the iliac bifurcation under a dissecting microscope [13]. The dissected aorta was placed in PBS, and fat and connective tissue adhering to the adventitia were removed as much as possible. The presence of atherosclerotic lesions in the aorta was measured using oil red O staining. Images were recorded using a digital camera (Coolpix 990; Nikon Corp, Tokyo, Japan), and lesion areas were analyzed using Image-J program. The aortic sinus was sectioned serially (5-μm intervals) and stained with hematoxylin and eosin (H&E). All images were digitized using a microscope (Olympus AX80; Olympus Optical, Tokyo, Japan) equipped with a high-resolution camera (Nikon D2X; Nikon).

### Analysis of serum lipids

Serum total cholesterol (TC), triacylglycerol (TG) were measured using enzymatic reagent kits (Biosino, Beijing, China), while high-density lipoprotein cholesterol (HDL-c) was detected by phosphotungstic acid (PTA)-Mg^2+^ precipitation method (Biosino, Beijing, China) and low-density lipoprotein cholesterol (LDL-c) was detected by polyvingel sulfate (PVS) sedimentation method (Biosino, Beijing, China). Plasma lipoproteins were fractionated by fast-protein liquid chromatography (FPLC) with slight modification according to Sips et al. [14]. Briefly, serum (100 μL) was subjected to gel-filtration chromatography using Superdex 200 10/300 GL (GE Healthcare Bio-Sciences AB, Uppsala, Sweden). Fractions (0.20 ml) were collected, and then cholesterol and TG concentrations were measured in the even-numbered fractions.

### Analysis of bile acid profiles using LC–MS

The bile acids were extracted from serum, bile, and liver by protein precipitation using ice-cold acetonitrile. Simply, 1 mL of ice-cold acetonitrile was added to 100 μL serum, 100-fold diluted bile and liver homogenate, and the sample was vortexed and centrifuged at 14800 rpm and 4 °C for 10 min. The supernatant was aspirated, evaporated under vacuum, reconstituted in 100 μL of MeOH and deionized water (85: 15, v: v), and centrifuged at 14800 rpm and 4 °C for 10 min. The supernatant was collected and 70 μL was injected into the LC–MS system for analysis with slight modification according to Yang et al. [15]. Liquid chromatographic separation and mass spectrometric detection were performed using the Agilent G6410 Triple Quad (QQQ) tandem mass spectrometer equipped with electrospray ionization (ESI) source, coupled with Agilent 1260 HPLC separation system (Agilent). The chromatographic separation was carried out using a ZORBAX SB-C18 analytical column (Agilent, Palo Alto, Calif., U.S.A.; 250 × 4.6 mm, i.d.; 5 μm). The mobile phase consisted of methanol (A) and 5 mmol/L ammonium acetate in aqueous solution (B) containing 0.1% formic acid at a total flow rate of 0.5 mL/min. The gradient profile for the LC pumps under the final chromatography conditions are as follows: 0 min, 10:90; 1.4 min, 50:50; 2.8 min, 65:35; 4.2 min, 70:30; 8.4 min, 75:25; 15.4 min, 80:20; 28 min, 92:8; 38 min, 93:7; 56 min, 93:7; 60 min, 10:90. The sample volume injected was 10 μL. ESI in the mass spectrometer was performed with the following parameters: ionization, negative ion mode; capillary voltage, 3.5 kV; atomizing voltage (N2), 40 psi; ion source temperature, 300 °C; drying gas (N2) flow rate, 10.0 L/min; drying gas temperature, 350 °C [16]. The mass range of FS mode was recorded from m/z 300 to 600. The data analysis was processed with Agilent Qualitative Analysis Workstation software.

### Quantitative real-time PCR

TRIzol reagent (Invitrogen, USA) was using to extract hepatic total RNA. Real-time PCRs were performed as described previously. Relative mRNA expression levels were determined by standard curve method normalized to 18S. Sequences of primers used were shown in Table 2.

**Table 2:** Primer sequences

Gene	Forward and reverse primers	Gene	Forward and reverse primers
*Nr1h4*	f: 5′-TCCAGGGTTTCAGACACTGG-3′ r: 5′-GCCGAACGAAGAAACATGG-3′	*Baat*	f: 5′-GGAAACCTGTTAGTTCTCAGGC-3′ r: 5′-GTGGACCCCCATATAGTCTCC-3′
*Nr0b2*	f: 5′-CGATCCTCTTCAACCCAGATG-3′ r: 5′- AGGGCTCCAAGACTTCACACA-3′	*Slc27a5*	f: 5′-ACCCTGGATCAGCTCCTGGAT-3′ r: 5′-GTTCTCAGCTAGCAGCTTGG-3′
*Cyp7a1*	f: 5′-AGCAACTAAACAACCTGCCAGTACTA-3′ r: 5′-GTCCGGATATTCAAGGATGCA-3′	*Abcc2*	f: 5′-GGATGGTGACTGTGGGCTGAT-3′ r: 5′-GGCTGTTCTCCCTTCTCATGG-3′
*Cyp7b1*	f: 5′- TAGCCCTCTTTCCTCCACTCATA-3′ r: 5′-GAACCGATCGAACCTAAATTCCT-3′	*Abcc3*	f: 5′-TCCCACTTTTCGGAGACAGTAAC-3′ r: 5′-ACTGAGGACCTTGAAGTCTTGGA-3′
*Cyp8b1*	f: 5′-GGCTGGCTTCCTGAGCTTATT-3′ r: 5′-ACTTCCTGAACAGCTCATCGG-3′	*Abcb11*	f: 5′-CTGCCAAGGATGCTAATGCA-3′ r: 5′-CGATGGCTACCCTTTGCTTCT-3′
*Cyp27a1*	f: 5′-GCCTCACCTATGGGATCTTCA-3′ r: 5′-TCAAAGCCTGACGCAGATG-3′	*Slc10a1*	f: 5′-ATGACCACCTGCTCCAGCTT-3′ r: 5′-GCCTTTGTAGGGCACCTTGT-3′
*18S*	f: 5′-CAGGCATTGCTGACAGGATG-3′ r: 5′-TGCTGATCCACATCTGCTGG-3′		

### Statistical analysis

The results are presented as mean ± standard error of mean (SEM). All data were subjected to analysis of variance using the SPSS software (version 18.0; SPSS Inc., Chicago, IL, USA). Differences between the means were tested by one-way ANOVA, and all detected significant differences were further evaluated by student’s t-test. The level of significance chosen was *P* < 0.05.

## Results

### TMAO increased fat mass in in apoE^−/−^ mice

As shown in Table 3, administration of TMAO for 8 weeks did not change body weight in apoE^−/−^ mice but result in a significant increase in fat mass, in which, visceral epididymal and inguinal white adipose tissue increased 22.8 and 25%, respectively. There were no differences in liver weight among the two groups.

**Table 3:** Growth parameters

	Control (*n* = 8)	TMAO (*n* = 8)
Food intake (g/day)	3.71 ± 0.02	3.77 ± 0.02
Initial BW (g)	26.3 ± 0.55	26.2 ± 0.53
Final BW (g)	25.5 ± 0.59	25.8 ± 0.49
Liver weight (g/100 g BW)	4.08 ± 0.08	4.23 ± 0.10
WAT weight (g/100 g BW)		
Epididymal WAT	1.14 ± 0.07	1.40 ± 0.06^*^
Perirenal WAT	0.30 ± 0.04	0.33 ± 0.03
Inguinal WAT	0.68 ± 0.06	0.85 ± 0.07^*^

Data are expressed as the mean ± SEM. BW, body weight; WAT, white adipose tissue. * *p* < 0.05, ** *p* < 0.01 vs. control, using Tukey’s test

### TMAO accelerated atherosclerosis

**Fig. 1:**
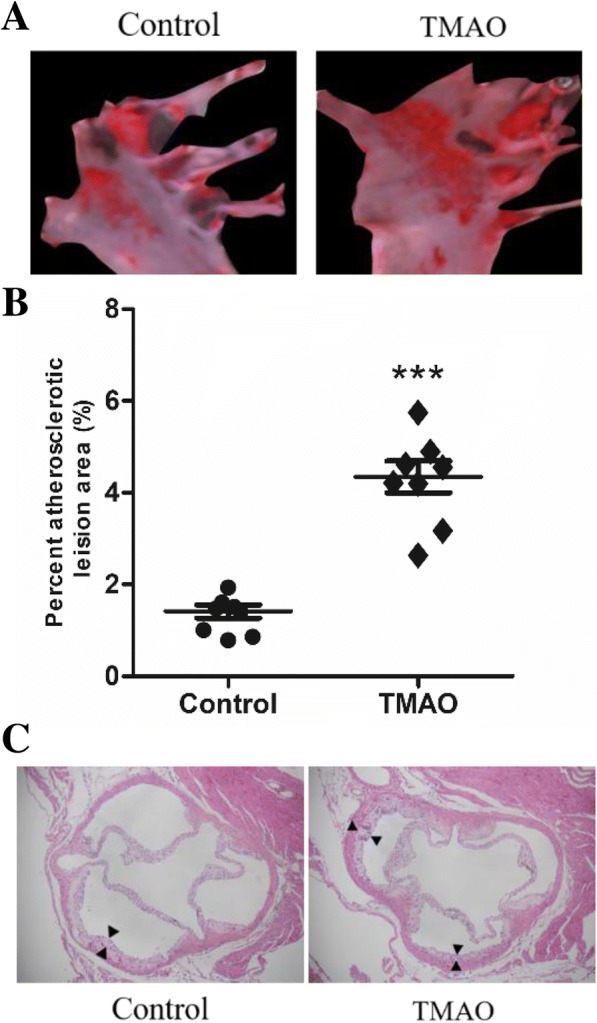
TMAO induced plaque progression in apoE^−/−^ mice (*n* = 8). **a** Atheromatous plaques in the aortas were visualized by oil red O (**b**) Atherosclerotic plaque area relative to total arterial wall area. **c** Representative photomicrographs of Hematoxylin & eosin (H&E)-stained, cross-sections of the aortic root showed medial thickness and formation of foam cell in the adventitia (40X magnification). *** *p* < 0.001 versus Control group

The total plaque areas in the aortas strongly increased 2-fold (*P* < 0.001) in TMAO administration mice compared with the control group (Fig. 1a and b). The tissue sections staining with H&E (Fig. 1c) showed unusual medial thickening and high infiltration of macrophage to the adventitia, and identified the accumulation of foam cells in the aortic wall in TMAO group.

### TMAO increased serum lipid concentrations

**Fig. 2:**
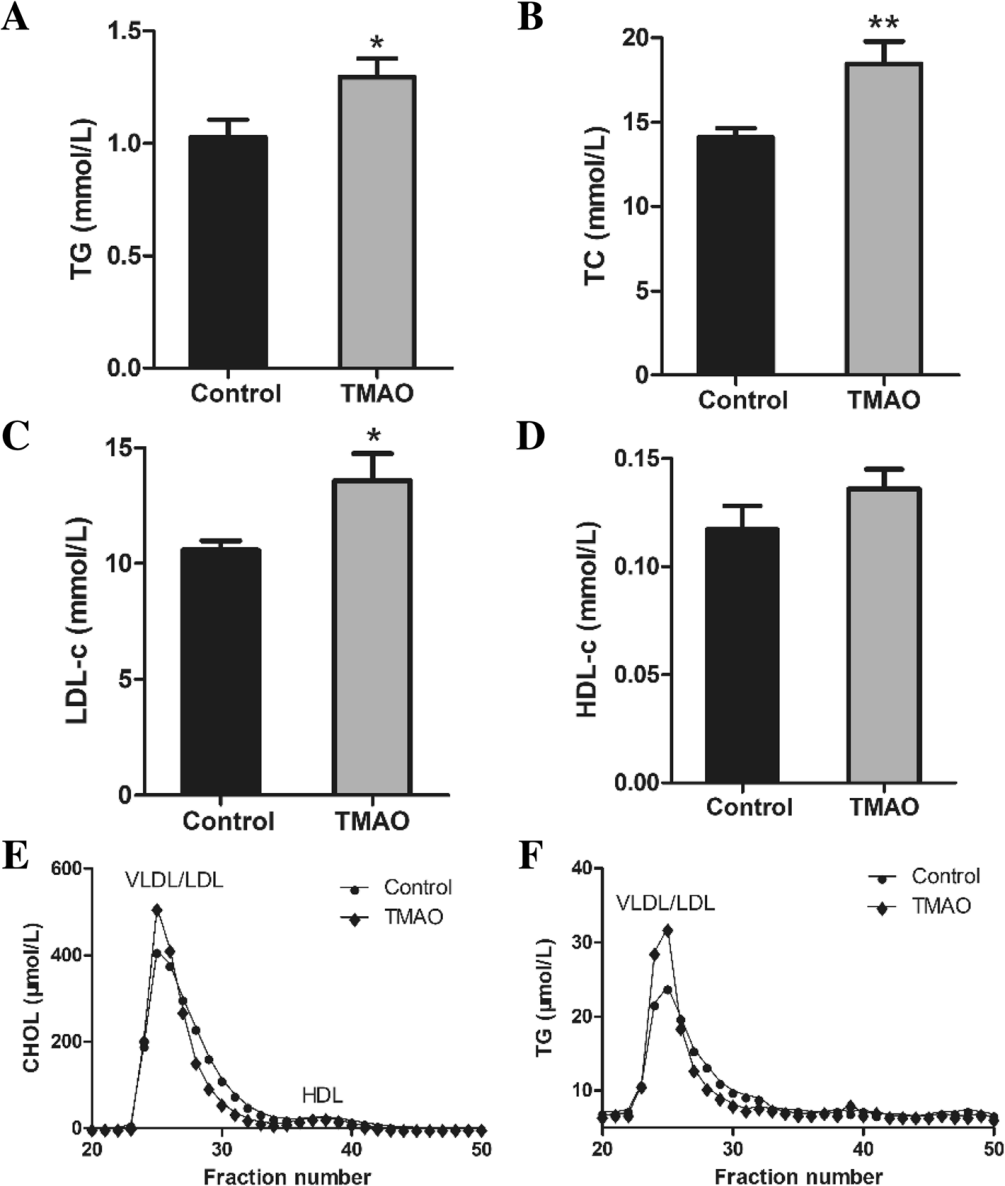
TMAO increased serum lipid levels. Serum TG (**a**), TC (**b**), LDL-c (**c**), HDL-c (**d**) were measured in apoE^−/−^ mice (n = 8). Plasma lipoproteins were fractionated by fast-protein liquid chromatography (FPLC). The serum (100 μL) was subjected to gel-filtration chromatography using Superdex 200 10/300 GL. Fractions (0.20 mL) were collected, and CHOL (**e**) and TG (**f**) concentrations were measured in the even-numbered fractions. TG, triglyceride; TC, total cholesterol; LDL-c, low-density lipoprotein; HDL-c, high-density lipoprotein; VLDL, very low-density lipoprotein; CHOL, cholesterol. Data are given as the mean ± SEM. *p < 0.05, **p < 0.01 versus Control group

Mice fed with TMAO exhibited significantly higher serum lipid. The levels of TG, TC, LDL-C in TMAO group were significantly increased by 25.5% (*P* = 0.044), 31.2% (*P* = 0.006), 28.3% (*P* = 0.032), respectively, compared with those of the control group (Fig. 2a-c). The concentration of HDL-C was unchanged by TMAO intervention (Fig. 2d). FPLC was used to fractionate the pooled plasma of male apoE^−/−^ mice. Results showed that the increased TC and TG were primarily associated with the high VLDL and LDL fraction in TMAO group (Fig. 2e-f).

### TMAO changed bile acid profiles

**Fig. 3:**
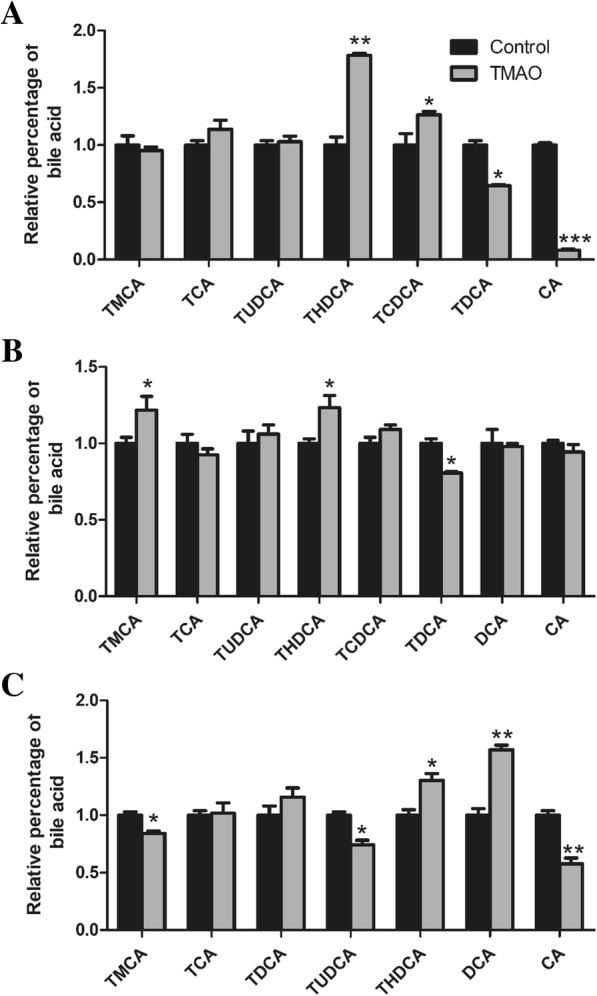
TMAO changed bile acid profiles. Bile acid species in apoE^−/−^ mice (*n* = 8) bile (**a**), liver (**b**) and serum (**c**) were quantified using LC-MS/MS. CA, cholic acid; DCA, deoxycholic acid; TCDCA, taurochenodeoxycholic acid; TCA, taurocholic acid; TDCA, taurodeoxycholic acid; THDCA, taurohyodeoxycholic acid; TUDCA, tauroursodeoxycholic acid; TMCA, tauromuricholic acid; Data are given as the mean ± SEM. *p < 0.05, **p < 0.01 versus Control group

To determine the fate of cholesterol, the bile acid profiles in the liver, bile and serum were measured by quantitative liquid chromatography coupled to mass spectrometry. As shown in Fig. 3a, the percentages of THDCA and TCDCA in bile were increased, while percentages of TDCA and CA were decreased by TMAO administration. In accordance with bile, TMAO induced high rate of THDCA and low rate of TDCA in liver (Fig. 3b). For serum sample, it was observed that THDCA and DCA in TMAO group presented at higher percentage than in control group (Fig. 3c). Notably, accordance with bile, the proportion of CA in serum was also significantly decreased by TMAO.

### TMAO inhibited hepatic bile acid synthesis

**Fig. 4:**
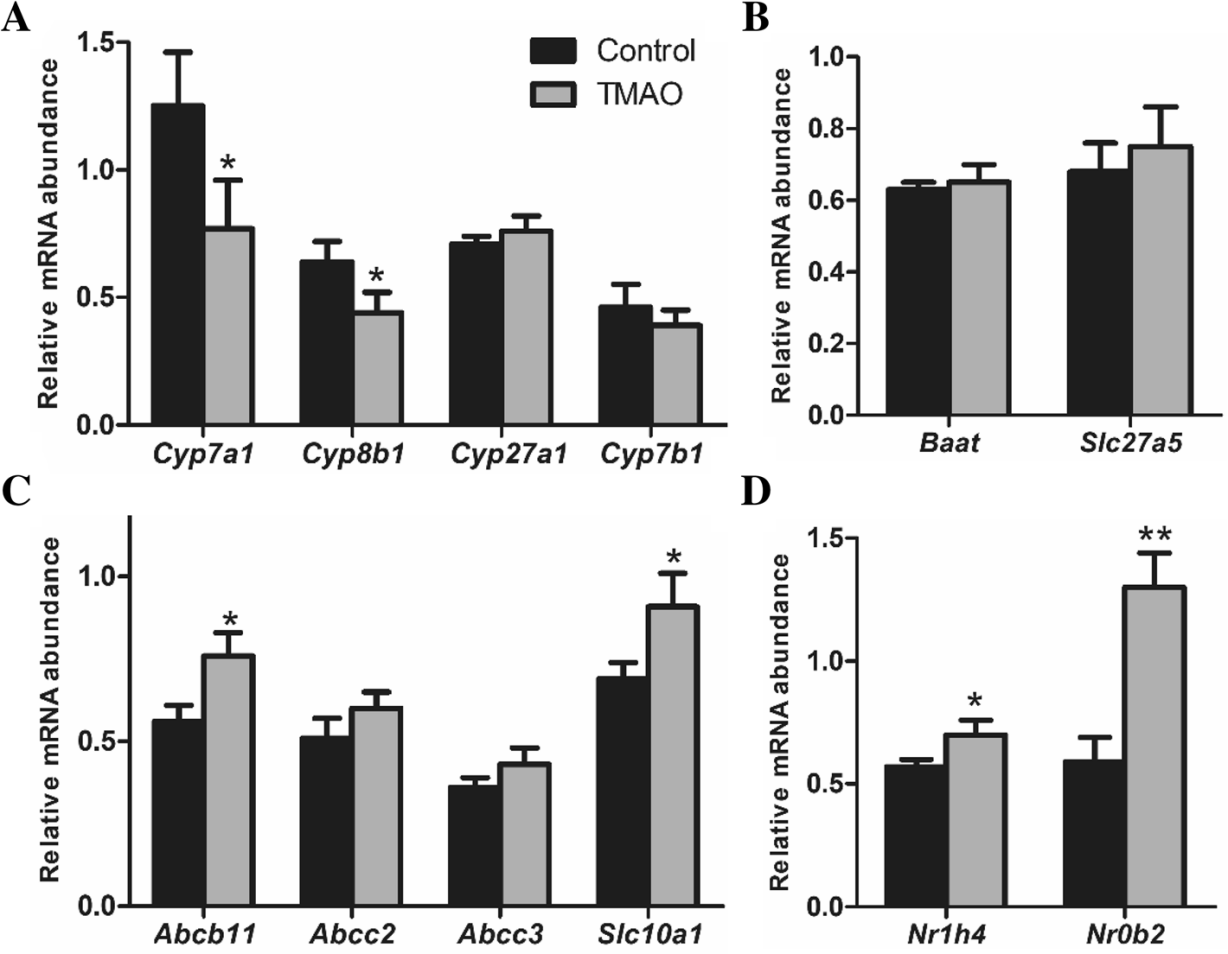
TMAO inhibited bile acid synthesis. Expression of genes involved in primary bile acid synthesis (**a**), conjugated bile acid synthesis (**b**), bile acid transport (**c**) and nuclear receptors in liver were determined by RT-qPCR. *p < 0.05, **p < 0.01 versus Control group

To determine the basis for TMAO-influenced hepatic bile acid synthesis, the mRNA analysis of liver tissue was performed to evaluate the expressions of both the classical and alternative bile acid synthesis pathways (Fig. 4a). Whereas *Cyp27a1* expression was unaltered, *Cyp7a1* expression was reduced 38.4% in TMAO intervention mice than in control diet mice (Fig. 4a), indicating specific downregulation of the classical bile acid synthesis pathway. Treatment with TMAO resulted in significant induction of *Abcb11* and *Slc10a1* expression, whereas other genes for bile acid transport (*Abcc2*, *Abcc3*) and conjugation (*Baat*, *Slc27a5*) were unaffected by TMAO. FXR plays a critical role in the regulation of bile acid synthesis and homeostasis. As shown in Fig. 4d, the expression of *Nr1h4* and *Nr0b2* were upregulated by TMAO administration.

## Discussion

Several studies have demonstrated that circulating TMAO levels were associated with obesity, type 2 diabetes and atherosclerosis [17–19]. It has been reported that FMO3 expression is linked to lipid and glucose metabolism. The decreased insulin levels in the livers of FMO3^−/−^ mice might result in reduced lipogenesis and further down-regulated PPARα expression [20]. Decreased KLF15, modulating gluconeogenesis [21], with decreased PPARα expression promoted inflammation in the livers of FMO3^−/−^ mice. Changes in bile acid metabolism could be linked to the inflammatory effects in liver, in which increased TNFα was proved to decrease the expression of Cyp7a1 through activation of the MAPK pathway [22]. Dietary choline, a precursor of TMAO, also reduced bile acid pool size in apoE^−/−^ mice and downregulated expression of CYP7A1 [23]. Based on the previous studies, it is not clear whether FMO3 or TMAO itself have an effect on bile acid metabolism. In the present study, we demonstrate the effects of directly dietray TMAO on bile acid metabolism, especially on bile acid profile.

A series of groundbreaking papers suggested that plasma TMAO concentration might be a biomarker of atherosclerosis [2, 24], and is closely related to dyslipidemia and impaired glucose tolerance [25, 26]. TMAO is formed from dietary TMA-containing nutrients, such as lecithin, choline, betaine, or carnitine, which can be metabolized in gut by bacterial lyases to release TMA, then the TMA was converted to TMAO by liver FMO3 [2, 4, 27]. In our previous study, it was observed that mice treatment with choline chloride through oral gavage or trimethylammonium chloride by intraperitoneal injection would increase the plasma TMAO level [28]. Wang et al. reported that apoE^−/−^ mice fed with diets rich in either choline (0.5% or 1% wt/wt) or TMAO (0.12% wt/wt) for 20 weeks resulted in increased aortic root lesion size [2]. In accordance with these results, in the present study, apoE^−/−^ mice directly administrated with TMAO for 8 weeks also showed notable progression of atherosclerosis.

The elevated levels of serum cholesterol could aggravate atherosclerosis progression [29]. Herein, we further examined the serum lipid profiles in mice fed with TMAO diet leading to the AS. The results showed that TMAO intervention caused high serum cholesterol concentration in normal diet-fed mice, especially LCL-c. It has been reported that dietary supplementation with high TMAO (1.5% TMAO in water) for 8 weeks could cause the hyperlipidemia in normal diet-fed mice with elevated levels of serum TC, TG, and LDL-c [4]. However, our previous study showed that there were no differences in serum lipid levels between the high-fat diet mice with or without TMAO intervention [26]. In addition, the western diet-fed apoE^−/−^ mice expressing hCETP treated with L-carnitine, a TMAO precursor, did not show any differences in lipid content compared with controls [30]. The observed lipid changes may be due to altered response to the diet with high fat or normal fat, and the hypothesis needs to be investigated in the future research.

The hepatic conversion of cholesterol to bile acids and ultimate excretion into the feces represent the major route for excess cholesterol excretion that is important in whole body sterol homeostasis [31]. Disruption of normal bile acid synthesis and metabolism is associated with atherosclerosis. Several studies have demonstrated that circulating TMAO negatively related to bile acid pool size and inhibited bile acid synthesis by decreasing CYP7A1 expression. The discovery that specific bile acids differentially activate different nuclear receptors, farnesoid X receptor (FXR), pregnane X receptor (PXR) and vitamin D receptor (VDR) and one G-protein-coupled receptor (TGR5), identified bile acids as hormones that alter multiple metabolic pathways in many tissues [11]. Although the relative importance of individual BAs in regulating these processes is not completely clarified, several bile acid species, such as CDCA and its conjugated forms, has been identified as FXR agonist [9]. In the present study, the bile acid composition in bile, liver and serum were detected by LC-MS, and the results indicated that the relative proportion of TCDCA was higher in TMAO supplementary mice, which might contribute to FXR activation and further inhibit bile acids synthesis.

The primary bile acids, CA and CDCA, are synthesized in hepatocytes via the cytochrome P450 (CYP)- mediated oxidation of cholesterol [31]. Majority of CDCA is converted to α-muricholic acid (α-MCA) and β-MCA in liver. When bile acids excrete into intestine, β-MCA is 7α-dehydroxylated to form hyodeoxycholic acid (HDCA) [32]. In the present study, the proportion of THDCA was increased and TMCA was decreased in serum of the TMAO group, which indicated that dietary TMAO prone to promote the forming of HDCA from MCA (an antagonist of FXR) in intestinal. It has been reported that reduced levels of TMCAs could promote FXR-dependent FGF15 expression in ileum and further inhibited the hepatic expression of CYP7A1 [10]. Previous studies have shown that FXR activation induces SHP, thereby suppressing CYP7A1 expression and ultimately inhibiting BA synthesis [33]. Our data showed that the mRNA expressions of FXR (encode by *Nr1h4*) and SHP (encode by *Nr0b2*) were significantly upregulated by TMAO. Notably, the serum proportion of DCA, as FXR activator, was increased approximately 60% by TMAO compared to control group. Therefore, the alteration of bile acid composition might be the major cause of activation of FXR and SHP, further inhibiting bile acid synthesis by repressing the expression of *Cyp7a1.*

To the best of our knowledge, the gut microbiota plays a key role in the pathophysiology of TMAO-induced AS. Various studies investigated the gut microbiota and key enzymes mediating the formation of TMAO in vivo [23, 24]. Here, we focus on the effect of dietary TMAO on bile acid metabolism, suggesting markable changes of bile acid profiles in apoE^−/−^ mice. However, one crucial question is how TMAO influences cellular metabolism and whether this is direct or indirect. In vivo loss-function experiments demonstrated that Flavin Monooxygenase 3 (FMO3) appeared to act as an important regulatory switch integrating cholesterol balance and hepatic inflammatory responses through mechanisms independent of its enzymatic product TMAO [34]. Moreover, a recent paper reported that the gut microbiota could contribute to the conversion of TMAO to TMA in mice gut [35]. Therefore, it reminds us that TMAO maybe not the “culprit” and we should take attention on the direct effects of metabolite on mediating the cellular metabolism.

## Conclusions

These findings suggested that TMAO accelerated aortic lesion formation in apoE^−/−^ mice by altering bile acid profiles, further activating nuclear receptor FXR and SHP to inhibit bile acid synthesis by reducing *Cyp7a1* expression.

## Abbreviations

CA: Cholic acid
CDCA: Chenodeoxycholic acid
CHOL: Cholesterol
DCA: Deoxycholic acid
FPLC: Fast-protein liquid chromatography
FXR: Farnesoid X receptor
H&E: Hematoxylin and eosin
HDL-c: High-density lipoprotein cholesterol
LCA: Lithocholic acid
LC-MS: Liquid chromatographic separation and mass spectrometric detection
LDL-c: Low-density lipoprotein cholesterol
SHP: Heterodimer partner
TC: Total cholesterol
TCA: Taurocholic acid
TCDCA: Taurochenodeoxycholic acid
TDCA: Taurodeoxycholic acid
TG: Triglyceride
THDCA: Taurohyodeoxycholic acid
TMAO: Trimethylamine-N-oxide
TMCA: Tauromuricholic acid
TUDCA: Tauroursodeoxycholic acid
VLDL: Very low-density lipoprotein
